# Phenomenological Modeling of Shape Memory Alloys: A Review of Macroscopic Approaches

**DOI:** 10.3390/mi16111300

**Published:** 2025-11-20

**Authors:** Girolamo Costanza, Maria Elisa Tata, Saeed Danaee Barforooshi

**Affiliations:** Industrial Engineering Department, University of Rome Tor Vergata, 00133 Rome, Italy; elisa.tata@uniroma2.it (M.E.T.); saeed.danaeebarforooshi@alumni.uniroma2.eu (S.D.B.)

**Keywords:** shape memory alloys, phenomenological models, constitutive models, thermomechanical coupling, hysteresis and cyclic behavior, rate-dependent effect, internal variables

## Abstract

Shape Memory Alloys (SMAs) have unique thermomechanical properties, including superelasticity and the shape memory effect, which has led them to be used in a wide range of applications, from biomedical devices to aerospace and civil engineering structures. These behaviors have been addressed by phenomenological models, which represent them by simply establishing stress–strain and transformation characteristics without accounting for the microstructure. In this review article, the main phenomenological modeling examples are categorized and compared, including the main principles of operation, predictions, and limitations under operating thermomechanical loading conditions. In addition, the growing use of SMAs, especially in actuation, damping, vibration control, and energy harvesting, is explored, and the incorporation of modeling frameworks into optimization activities is discussed. The final part of the review deals with open challenges and future research directions, consisting of the development of models that more accurately predict SMAs under cyclic and/or non-proportional loading, a more robust association with commercial computational tools, and exploring the use of SMAs in new interdisciplinary areas. By bridging modeling approaches to application-based concepts, a platform is provided for the advancement of both the scientific development and practical use of shape memory alloys.

## 1. Introduction

Shape memory alloys (SMAs) are a category of metallic materials that exhibit a reversible martensitic phase transformation that manifests itself in two different thermomechanical phenomena: the shape memory effect (SME) and superelasticity (or pseudoelasticity). The origin of both phenomena lies in a diffusionless transformation in a solid state from a high-temperature phase with high symmetry, austenite, to a low-temperature phase with low symmetry, martensite [1,2].

In the shape memory effect, a deformation in the martensitic phase of a material can be brought back to the undeformed shape by heating it above a critical transformation temperature and transforming it back into austenite [3]. On the other hand, superelasticity occurs when deformation takes place at a temperature above the final austenite temperature. In such a situation, high strains (usually up to 6–8%) are recovered immediately and reversibly while unloading by a stress-induced martensitic transformation and a subsequent reverse transformation [4,5]. Recovery is achieved without residual deformation, which makes SMAs particularly useful for applications in actuators, damping, and biomedical applications.

Martensitic transformation exhibits both diffusionless and displacement features as it involves cooperative shearing of atoms as opposed to diffusion over large areas, which explains the potential for reversibility and rapid response [6]. The thermomechanical coupling associated with this transformation creates a strong hysteresis in the stress and temperature-strain curves that must be accounted for by any modeling method that allows accurate simulation of SMA behavior [7].

Shape memory alloys can be formed from multiple compositions, with Ni-Ti-based alloys evidently the most researched due to their high performance and stability in thermomechanical applications. Other commonly studied shape memory alloy systems include Cu alloys (e.g., Cu–Zn–Al and Cu–Al–Ni) and Fe alloys (e.g., Fe–Mn–Si) that are useful in civil and structural settings. This review article will focus predominantly on the use of Ni–Ti–based shape memory alloys since they are the most used in phenomenological constitutive modeling and engineering applications.

The advanced functional properties of shape memory alloys allow them to be utilized in various fields of engineering. In medicine, self-expanding stents, orthodontic arch wires, bone anchors, and minimally invasive surgical tools incorporate SMAs due to their biological compatibility and corrosion resistance along with their capability to provide a constant stress within a certain range of loads [8,9]. SMA components are widely applied in aerospace engineering within morphing structures, deployable actuators, aerodynamic control surfaces, and vibration dampers because the material’s high working power density is advantageous along with its ability to deform reversibly [10,11]. In robotics, SMAs are appealing for the use in miniature-sized actuators and soft robots’ joints as well as artificial muscles owing to their silent operation, low weight, and high energy density in comparison to classical actuators [4,12].

To optimally suit these applications requires a precise model due to the complex thermomechanical behavior of SMAs. Accurate simulation helps in prediction of phase change attributes, the evolution of stress–strain–temperature and hysteresis for varying operating conditions. This governs the design, simulation, as well as real-time control in smart devices and structures [13,14].

Modeling approaches for SMAs can be classified very broadly into:

Micromechanical models that encompass the crystallographic and grain-level phenomena [15,16,17,18,19,20,21,22,23,24,25,26];

Thermodynamic models that take fundamental energy principles to derive constitutive behavior [7,27,28,29,30,31,32,33,34];

And phenomenological (macroscopic) models that support generalized observations through empirical rules and simplified formulations, capturing material responses without explicitly resolving micro-scale features.

Among these, phenomenological models, are particularly desirable for engineering purposes because of their low computational cost and ease of implementation. These models reproduce stress–strain–temperature responses by introducing internal variables such as martensitic volume fraction, transformation strain, and hardening parameters. While these phenomenological models cannot be interpreted microstructurally, they have demonstrated significant success in capturing key behaviors such as hysteresis, phase transformation paths, and rate dependence when they are extended in a considered way.

In recent times, many models have been created in three-dimensional (3D) space to demonstrate the complex thermomechanical behavior of SMAs. There are notable examples in the literature, including that of Boyd and Lagoudas [35], Ivshin and Pence [36], and Graesser and Cozzarelli [37]. Each of these 3D models attempts to evolve highly nonlinear stress states, phase transformations, and coupling of mechanical and thermal fields. However, although compendious in extent, they have limited acceptance in engineering practice.

One of the greatest limitations of the 3D models is their complexity. They include various internal variables, multiple transformation criteria, and consistent thermodynamic formulations that require extensive calibration. The effort to develop multiple internal variables causes them to be computationally expensive and more difficult to implement, particularly when speed is needed for control systems or actuator development in real-time conditions or as embedded applications.

These models could be reduced to one-dimensional (1D) form to improve implementation, but this diminishes the main benefit of the full 3D formulation. Additionally, for applications that inherently undergo uniaxial loading conditions (i.e., SMA wires, strips, and rods), it is often unnecessary or inefficient to develop uniaxial models. In an ideal situation, it would be a more reasonable approach to evaluate the output of 1D phenomenological models.

A notable advantage of 1D models is they are based on engineering relevant parameters, critical transformation stresses, maximum transformation strains, and characteristic temperatures. Thus, the parameters are easily derived from routine experimental tests (i.e., uniaxial tensile tests or thermal cycle tests) and rather easily calibrated into the model. Furthermore, 1D models yield a starting point that provides a less complex set of mathematics and is therefore faster to simulate, and when deemed necessary, for example, in a smart structure, automation, robotics, or control-oriented modeling, this is a strong fit. These models provide reasonable capabilities but run into problems such as non-proportional loading, strain–rate dependence, and complicated thermal cycling. New developments seek to resolve these problems through phase-dependent stiffness, revised hardening laws, rate dependence, or even nonlinear evolution functions, while preserving the simplicity of a 1D structure [38]. Nevertheless, unidimensional models are the basis of multiaxial and thermo-mechanically coupled SMA modeling. They are also the basis for many SMA applications involving wires, strips, and rods where the principal mode of deformation is axial. Due to their reduced form, they provide a useful trade-off between physical realism and computational efficiency and have become very useful in system-level simulations, control design, and educational modeling environments [39,40].

The early development of 1D phenomenological models began with the pioneering work of Tanaka [41]. He developed a framework to correlate transformation strain to the martensitic fraction evolution, based on some thermodynamic driving forces. This framework was improved upon by Liang and Rogers [42], who improved the temperature dependence and provided kinetic laws for the phase transformation. The contribution of Brinson [43] introduced discrete tracking of stress- and temperature-induced martensite with separate transformation strains to allow for greater flexibility in the model. These models evolved into a substantial model development, many of which are referenced and modified in contemporary models.

This review intends to present a systematic and comparative review of phenomenological models developed for 1D SMA behavior. By providing an overview of both classical and modern phenomenological models, this review seeks to assist researchers and engineers in selecting or developing an appropriate tool for simulating SMA behavior in different systems.

## 2. Fundamental 1D Models of Shape Memory Alloys

### 2.1. Tanaka Model

The Tanaka model is one of the earliest phenomenological models to characterize the macroscopic thermomechanical response of shape memory alloys, proposed by Kenji Tanaka in 1986 [41]. The Tanaka model acts as a 1D constitutive model relating the internal transformation mechanism of the material to the observable macroscopic response using internal variables. The model is based on a thermodynamic framework and was developed as a simplified means of describing the shape memory effect and superelasticity for uniaxial loading.

The defining variable for Tanaka is the martensitic volume fraction (ξ), an internal state variable that is defined as the fraction of the material that has transformed from the high-temperature austenite phase to the low-temperature martensite phase.

The constitutive relationship links the current stress σ to strain ε, temperature T, and the martensite volume fraction ξ. The stress relationship can be expressed as follows [38]:

(1) $$\sigma -\sigma_{0}=E\left(\xi \right)(\varepsilon -\varepsilon_{0})+\theta \left(T-T_{0}\right)+\Omega (\xi )(\xi -\xi_{0})$$

where $\sigma_{0}$, $\varepsilon_{0}$, $T_{0}$, and $\xi_{0}$ are the initial stress, strain, martensitic fraction, and temperature of the material, respectively.

The modulus of elasticity $E\left(\xi \right)$ is also not constant and is given as linearly varying with ξ to account for the change in stiffness due to phase transformation as follows [38]:

(2) $$E\left(\xi \right)=E_{A}+\xi (E_{M}-E_{A})$$

where $E_{A}$ and $E_{M}$ are the elastic moduli of the austenitic and martensitic phases, respectively.

To account for the effects of strain due to the transformation, he used a transformation-influence coefficient, denoted as Ω(ξ), which is defined as follows [38]:

(3) $$\Omega \left(\xi \right)=-\varepsilon_{L}E\left(\xi \right)$$

where $\varepsilon_{L}$ denotes the maximum recoverable transformation strain. This term describes the internal stress given the growing martensite content.

The martensite fraction evolves based on exponential functions, describing the kinetics of forward and reverse phase transformation. This kinetics equation describes the fraction of phase transformation as an exponential function of the stress and temperature as follows [38]:

(4) $$\left\{\begin{array}{c} \xi_{M\to A}=\exp\left[a_{A}\left(T-A_{s}\right)+b_{A}\sigma \right]\\ \xi_{A\to M}=1-\exp\left[a_{M}\left(T-M_{s}\right)+b_{M}\sigma \right] \end{array}\right.$$

where $a_{A}$, $a_{M}$, $b_{A}$, and $b_{M}$ are material constants, respectively, relate to the $A_{s}$, $A_{f}$, $M_{s}$, and $M_{f}$.

### 2.2. Liang and Rogers Model

A constitutive framework similar to Tanaka model has been developed, which has essentially the same formulation for stress–strain relations. The main difference lies in the formulation of phase transformation kinetics. Rather than using a linear or exponential dependence, this transformation will use a cosine-based relation to describe the evolving martensitic volume fraction. Speaking of which, the model that will be used will describe transformation behavior as a function of both applied stress and temperature and adds the benefit of a smooth and continuous description of the phase change process.

(5) $$\left\{\begin{array}{c} \xi_{A\to M}=\frac{1-\xi_{A}}{2}\cos\left[a_{M}\left(T-M_{f}\right)+b_{M}\sigma \right]+\frac{1+\xi_{A}}{2}\;\;\;\;\;\;\;\;\;\;\\ for\;\;\;T>M_{f}\;\;\;and\;\;c_{M}\left(T-M_{s}\right)<\sigma <c_{M}\left(T-M_{f}\right)\\ \xi_{M\to A}=\frac{\xi_{M}}{2}\cos\left[a_{A}\left(T-A_{s}\right)+b_{A}\sigma +1\right]\\ for\;\;\;T>A_{s}\;\;\;and\;\;c_{A}\left(T-A_{f}\right)<\sigma <c_{A}\left(T-A_{s}\right) \end{array}\right.$$

In which $a_{A}$, $a_{M}$, $b_{A}$, and $b_{M}$ are material constants [42].

### 2.3. Brinson Model

In 1993, Brinson [43] introduced a one-dimensional model for SMAs, building upon the foundational work of Tanaka and Liang. While adopting Tanaka’s constitutive equation and adhering to the same thermodynamic framework, Brinson’s formulation addressed a key limitation of the earlier Liang model. The Liang model could not address the shape memory effect associated with martensite detwinning at temperatures below the martensite finish temperature. This shortcoming was effectively resolved in Brinson’s approach. This model separates two mechanisms of martensitic transformation by decomposing the total martensite volume fraction into a stress-induced and a temperature-induced volume fraction component as follows [43]:

(6) $$\xi =\xi_{s}+\xi_{T}$$

In which $\xi_{s}$ and $\xi_{T}$ are stress-induced and a temperature-induced volume fractions, respectively.

Phase transformation kinetics in Brinson model are presented as follows [43]:

(7) $$\left\{\begin{array}{c} \xi =\frac{\xi_{0}}{2}\cos\left[a_{A}\left(T-A_{s}-\frac{\sigma }{c_{A}}\right)\right]+\frac{\xi_{0}}{2}\;\;\;\;\;\;\;\;\;\;\\ for\;\;c_{A}\left(T-A_{f}\right)<\sigma <c_{A}\left(T-A_{s}\right)\\ \xi =\frac{1-\xi_{0}}{2}\cos\left[a_{M}\left(T-M_{f}-\frac{\sigma }{c_{M}}\right)\right]+\frac{{1+\xi }_{0}}{2}\\ for\;\;c_{M}\left(T-M_{s}\right)<\sigma <c_{M}\left(T-M_{f}\right) \end{array}\right.$$

Based on separation of martensite fraction, the constitutive equation was also modified accordingly as follows [43]:

(8) $$\dot{\sigma }=D\dot{\varepsilon }+\Theta \dot{T}+\Omega {\dot{\xi }}_{s}k$$

#### Additional Influential Models Related to 1D Formulations

In addition to the three classical 1D phenomenological models, several other influential formulations have played a vital role in development of SMA constitutive modeling. Among them, the Boyd–Lagoudas [35] and Auricchio [44] models have received substantial attention in the literature and are regarded as milestones in developing SMA constitutive modeling due to their strong thermodynamic foundations and comprehensive treatment of phase-transformation behavior. Specifically, the Boyd-Lagoudas model has been recognized as the first model to create a rigorous thermodynamic framework to describe martensitic volume fraction evolution and transformation-induced strain. Auricchio’s formulation followed the Boyd–Lagoudas model to provide enhanced numerical stability in formulating the SMA constitutive model, yet simultaneously incorporated transformation-induced plasticity and captured the main features of the SMA cyclic response. While the Boyd–Lagoudas and the Auricchio models were originally 3D models, they nevertheless, have important influence on 1D uniaxial model formulation and on the advancement of SMA constitutive modeling in general.

In addition to classical constitutive models, researchers have attempted to model SMA behavior into frameworks that facilitate coupling of the thermomechanical response, control action, and minor hysteresis. Ikuta et al. [45] proposed an SMA actuator model based on electrical resistance feedback, which allowed SMA wires activated by heat to be controlled in real time for motion application in compact robotic applications. This work introduced the concept of feedback via intrinsic resistance change and used this variable to model actuation, sensing, and control. Madill and Wang [46] developed an SMA actuator model by studying the dynamic behavior of positioning systems using SMAs and explored closed-loop stability with L2-gain techniques. Their approach aimed to integrate constitutive nonlinearities relevant to control in the design process of an SMA actuator. Most recently, Mitrev et al. [47] used a thermomechanical approach to model major and minor hysteresis loops while subjected to thermal and mechanical load variances. This was one of the limitations of classical phenomenological approaches, which assume transformations are complete at each reset of the cycle. As these investigations do not strictly have to do with fundamental constitutive models, they provide all significant alternative approaches to critical SMA modeling and show not only the importance of minor transformation cycles at the application level, but also the importance of sensing and control in use.

To make it more clear, when an SMA is subjected to thermomechanical cycling, and the forward (or reverse) phase transformation is not completely achieved, minor hysteresis loops develop as a result of partial transformation between austenite and martensite. These loops typically occur when the applied thermal or mechanical load remains within the transformation temperature intervals—i.e., between $A_{s}$ and $A_{f}$ during heating, and between $M_{s}$ and $M_{f}$ during cooling—causing only a portion of the material to undergo transformation. As a result, the stress–strain or strain–temperature response exhibits internal sub-loops distinct from the major hysteresis loop that corresponds to a complete transformation. Minor hysteresis is most relevant for SMA components subjected to small-amplitude thermal cycling, vibration-based actuation, or when a system undergoes fatigue, especially when feedback-controlled, where the material frequently operates in a partial transformation region. Classical 1D models largely deal with major transformation paths and do not capture the internal sub-loop behavior that arises when partial cycle models are considered, which may lead to inaccurate predictions of actuator performance, energy dissipation, and fatigue accumulation. The recognition and explicit accounting for minor hysteresis behavior have been increasingly recognized as important for realistic modeling of SMA components, particularly for applications such as micro-actuators, adaptive structures, biomedical devices, and energy harvesters.

## 3. Comparative Evaluation of Fundamental 1D Phenomenological SMA Models

A comprehensive and detailed examination of the Tanaka, Liang–Rogers, and Brinson models, reveals the evolution in phenomenological modeling of shape memory alloys from basic constitutive types to appropriate modeling structures that can more fully characterize complicated thermomechanical processes. While the Tanaka, Liang-Rogers, and Brinson models are designed to describe the global relationship between stress, strain, and temperature in an SMA, there are significant differences between the models regarding mathematical formulations, internal state variables, patterns of phase transformations, applicability, and modeling assumptions.

Table 1 compares the relevant aspects, benefits, and drawbacks of the basic 1D phenomenological models for SMAs: the Tanaka, Liang–Rogers, and Brinson models. This comparison allows us to understand each model’s main assumption, mathematics, number of parameters, and intended use.

The three 1D phenomenological models, Tanaka, Liang–Rogers, and Brinson, represent a milestone for SMA modeling on a macroscopic level. They all attempt to provide a mathematically simple description of the shape memory effect and superelasticity, but they differ in how they model the kinetics of phase transformation, internal variables, and predictive capability for real applications.

Tanaka’s model is, and always will be, the historical benchmark. Tanaka’s model allowed the number of natural variables to connect the microscopic phase transformation to macroscopic stress–strain–temperature behavior. The model includes a constitutive equation that contains a stress–strain relation, where the basic variables are linked to a temperature term and an internal variable term that has a linearly varying elastic modulus to capture the change in phase fraction. The exponential kinetic law constructed for forward and reverse phase transformations is also mathematically simple for researchers to implement; however, it should be noted that purely mechanical formulation may cause abrupt and non-hysteretic transitions, unlike experimental results where hysteresis loops are typically smooth. Although Tanaka’s model is limited to elastic moduli ($E_{A},\;E_{M}$), transformation strain, transformation temperatures ($M_{s},\;M_{f},\;A_{s},\;A_{f}$), and a couple of coefficients for the kinetics, it remains relatively easy to calibrate using uniaxial tensile data or thermal cycling data.

The Liang–Rogers model improves upon Tanaka’s model by changing the way that phase transformation kinetics is taken into account. Instead of an exponential equation, the Liang–Rogers model includes cosine-based kinetic equations to create a smoother and more continuous phase transformation from martensite to austenite. This development improves the agreement with experimental stress–strain curves, in particular for partial loading-unloading cycles that have a gradual phase transformation compared to instantaneous. The constitutive mechanics remain fundamentally the same as Tanaka’s since both models incorporate an internal variable and a linear elastic modulus variation; the smoother transformation kinetics reduce numerical instability and make the model more robust for multiple cycling. The only additional consideration in the Liang–Rogers model is further parameters to describe the shape of the cosine functions.

Brinson’s model provides a solution to one of the critical limitations of phenomenological SMA modeling, the inability to differentiate between stress-induced martensitic transformation (i.e., superelasticity) and temperature-induced transformation (i.e., shape memory effect). Brinson proposed to divide the total martensitic fraction into two contributions: for stress-induced martensite and for temperature-induced martensite. This division enables the model to reproduce progressively complex mechanisms, such as detwinning martensite at low temperatures and partial transformation during combined thermal–mechanical cycles. The phase transformation kinetics are still based on the cosine approach, but they are now defined separately for each martensitic fraction, which will increase the number of material parameters that must be calibrated. Likewise, the constitutive relation is now developed with the separate evolution of $\xi_{s}$ and $\xi_{T}$ making it more capable of replicating hysteresis loops and cyclic loading behavior, but it comes with a cost which includes additional calibration steps and increased computational time and effort.

In engineering simulations that involve practical applications, you must strike a balance between predictive power and simplicity. Tanaka’s model is still useful in simple design studies, as well as in modeling for control, and in contexts of teaching the principle of phase transformation where an accurate model is not requested. The input parameters are limited, usually requiring only a standard uniaxial tensile test to specify, and can be implemented very quickly, making it reasonable to use in feasibility studies or where simple actuators are being constructed.

The drawback of each of these models is that they are fundamentally only one-dimensional. Each model will do a good job describing how SMA wires, strips, rods, and springs behave when subjected to uniaxial or primarily axial loading situations. These models are not intended to predict multi-axial complex stress states or phenomena like stress concentrations since these would be better described with more sophisticated 3D micromechanical or thermodynamic models.

None of these models take rate-dependent effects into full account, which can become significant in dynamic applications where there can be high rates of loading or thermal cycling. Although Brinson’s formulation can be seen as an incremental improvement by better describing detwinning and some partial phase transformations, the kinetic rules are still phenomenological, describing an approximation instead of describing microstructure. Therefore, if the task is of high-fidelity importance, where local stress distributions or reorientation of grains or factors in fatigue behavior are required, more advanced or hybrid models may be required.

The models represent a continuum of trade-offs for computational efficiency, model parameterization effort, and physical realism:

- Tanaka: Best suited for conceptual design and simple feasibility assessment.
- Liang–Rogers: Better suited for the case of relying on repeated or smooth cycling where numerical stability is important.
- Brinson: Most flexible of the three for applications requiring a combination of thermal and stress loading, realistic hysteresis, and cyclic actuation.

Having a grasp of these trade-offs allows researchers and engineers to choose the best tool for their SMA system, depending on whether the designed aim is fast simulation for control design or more realistic prediction of actual device behavior.

## 4. Recent Developments and Trends in 1D Phenomenological Modeling

In the last twenty years, much has been accomplished to advance phenomenological modeling of shape memory alloys. In this section, we summarize the recent accomplishments from two interconnected perspectives: (1) theoretical and constitutive modeling of one-dimensional SMA behavior, which may include thermomechanical and rate effects, and (2) functional applications and structural applications, including actuators, springs, beams, and dynamic systems.

Our intention is twofold; first, we aim to summarize how SMA modeling approaches have changed through the addition of more physical phenomena, and through the application of concepts in the field of engineering. The summary will ultimately illustrate the extent of improvements in 1D macroscopic models to explain complex characteristics such as rate dependence, functional degradation, torsional response, cyclic effects, and the trajectory to include these models into a realistic structural analysis and actuator design.

The subsections ahead will present the new developments in phenomenological and constitutive modeling first, and applications will follow. This bifocal perspective is useful for elucidating both model development and real-world use and application, perspective to identify limitations and opportunities for current and future SMA research.

### 4.1. Constitutive and Theoretical Modeling Advances

Over the past two decades, a number of phenomenological constitutive models describing shape memory alloys have been developed, moving from relatively simple models focused on capturing pseudoelasticity and thermal cycling, to increasingly complex models that have incorporated additional factors, including temporally dependent rate effects, functional degradation, and loading conditions.

Auricchio, and Reali [44] developed a complex model in 1D form that introduced permanent inelasticity, allowing for saturating and non-saturating evolution laws, with a more realistic depiction of residual strains after cyclic thermomechanical loading. Zhu and Zhang [48] recognized the role of strain rate in determining the mechanical response for NiTi wires. To incorporate rate effects, Zhu and Zhang used mechanical laws to represent thermodynamics, energy balance laws, and phase transformation kinetics to capture thermal effects on phase transformation, while accounting for displacement rate and thus loading conditions. Experimental tests on superelastic NiTi wires over a wide range of loading rates (10^−4^ to 0.7 $S^{-1}$) verified the model’s effectiveness in predicting hysteresis reduction, transformation plateau variations, and temperature changes. The ability of this framework to demonstrate equivalent ratios of damping and stiffness under cyclic loading is an important part of the analysis for dynamic analysis of large structural systems containing SMA devices.

Auricchio, Fugazza, and DesRoches [49] introduced a viscous rate-dependent model that relied on internal martensite fractions, which could reproduce smooth transitions between elastic and transformation states. Their work has been particularly useful for dynamic applications.

**Figure 1:**
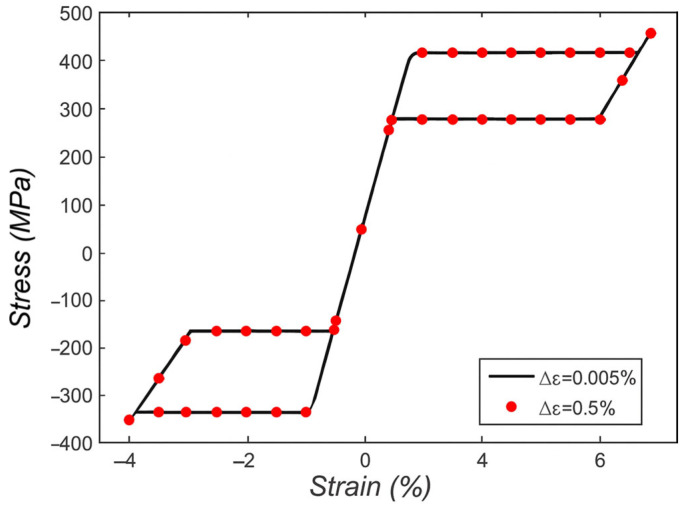
Strain-driven superelastic test (rearranged from [51]).

The multi-dimensional accuracy and computational efficiency problem was discussed by Evangelista, Marfia, and Sacco [50], who presented consistent 3D and 1D phenomenological models. These models enable a physical interpretation of material parameters, while reducing the computational cost during analyses of tensile, compressive, and bending loading. In addition, Auricchio, Reali, and Stefanelli [51] extended past 1D macroscopic models to consider tension-compression asymmetry, and elastic properties that are dependent on stress transformations, thereby, generating more realistic stress–strain loops with asymmetry with different responses. Figure 1 shows the stress–strain response in superelastic case, which proves the performance and robustness of the model in different strain step increments.

Another significant research effort emerged that centered on the rate-dependent response of SMAs in which thermomechanical coupling was explicit. This approach allowed them to better articulate the coupling between thermal and mechanical processes because it explicitly gave a thermomechanical coupling physics-based treatment to the rate dependence for SMAs. Previously proposed viscous-type mechanics were modified in a continuum mechanics framework into a phenomenological 1D constitutive model in which the energy equation coupling had terms that explicitly included and accounted for heat transfer effects. This was significant as it formed a more physically consistent and clearer representation of rate dependence, whereby the transformation stresses were functionally described not only in mechanical ways (i.e., stress and strain rate) but also involved a full interaction between a rate of mechanical loading and thermal exchanges associated with the environment. This development enabled comparisons between numerical simulations and experimental results across a range of loading rates, which also included thermal conditions. In the end, the model essentially captured cyclic responses and tension–compression asymmetry in a completely thermomechanical way, while acknowledging the role of plasticity on temperature changes with transformations. Overall, this work established a basis for discussing SMA rate dependency and advancing a broader understanding by clarifying the role of thermomechanical coupling on the macroscopic response of SMAs [52].

Without looking into some detail of Brinson’s past works, Khandan, Akbarzadeh, and Marfat [53] made some refinements to Brinson’s 1D model by addressing compatibility problems and developing consistent transformation tensor expressions, which provided a reasonable basis for modeling in the future.

In terms of the practical implementation, particularly for seismic and other dynamic applications, Qian et al. [54] presented a rate dependent SMA model with internal temperature variables to account for the effect of loading frequency, validated using quasi-static and dynamic cyclic tensile test data. The model demonstrated good agreement with experimental data and showed potential for SMA-based passive energy dissipation devices, extending one-dimensional models to dynamic and/or rate-dependent structural uses.

Frost et al. [55] developed a 1D model that accounts for the martensitic transformation, detwinning, and partial loading cycles for NiTi wires, and then implemented it as a UMAT for use in Abaqus; it was very flexible in modeling applications using a SMA-based device. The model accurately simulated full and partial martensite–austenite transformation, and its flexibility in being adaptable to different NiTi materials by altering experimentally measurable input parameters allows for the model to be used as a finite element simulation tool, and for wire-based SMA use.

Ryu et al. [56] modeled SMA springs in which they reduced the complex 3D loading applied to the spring into an effective 1D axial deformation model, including Young’s and shear moduli that were subsequently corrected for use in practical engineering design. Marfia and Rizzoni [57] developed a three-phase 1D constitutive model which accounted for tensile martensite, compressive martensite, and austenite to simulate the pseudoelasticity, tension-compressive asymmetry occurrence, and bending behaviors, while simultaneously simplifying the multivariant martensite reorientation process. A finite element implementation with a two-node beam element showed great agreement with experimental observations. This study also recognized the necessity of considering the distinct elastic properties of the different SMA phases (austenite and martensite) and showed it was valid to use Voigt and Reuss homogenization techniques. This research represents a significant advance over previous 1D modeling efforts, allowing realistic simulations of SMA wires beams in complex thermo-mechanical load histories.

Oliveira, Savi, and Santos [58] were explicit in their justification of uncertainty quantification, which stems from the sensitivity analysis conducted on 1D SMA models they developed. They suggested that elastic moduli and temperature effects were major consequences of predictive uncertainty. Parametric analysis revealed that elastic moduli $E_{A}$ and $E_{M}$, and temperature, exerted the greatest influence on the response. Minor differences in modeling response were identified at the beginning of the phase transformation and at the transition temperature; however, the model sufficiently captured the overall nonlinear thermomechanical response of SMAs. This work has highlighted the critical role that uncertainty and sensitivity analysis play in validating SMA constitutive models and could potentially inform their use for more complex simulations.

**Figure 2:**
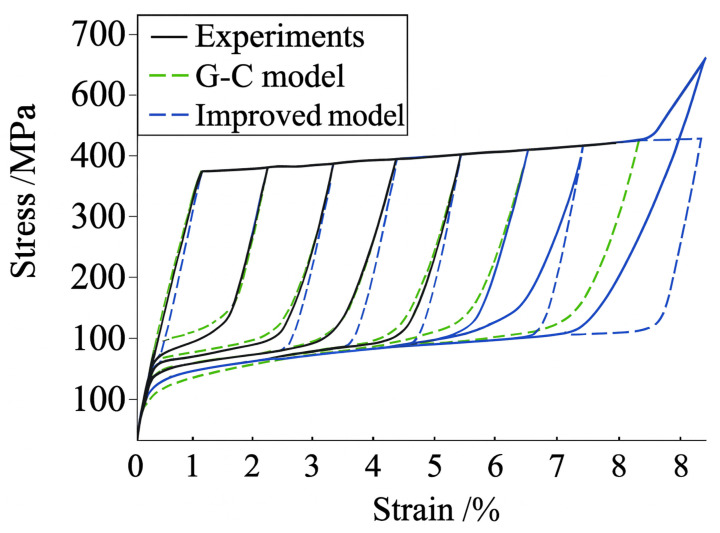
Comparison of the hysteretic curves between experiments, G-C model and improved G-C model (rearranged from [59]).

Liu et al. [59] enhanced the Graesser–Cozzarelli model by including strain–amplitude and strain–rate dependence, segmenting hysteretic responses into loading and unloading stages, providing fitted expressions for characteristic parameters, and facilitating realistic simulations for superelastic SMA wires used in vibration control systems. Experimental and simulated hysteresis of superelastic SMA wire is shown in Figure 2, which has excellent agreement between the improved model and the experimental one under a fixed strain rate but various strain amplitudes.

Lee and Jeon [60] proposed a phenomenological hysteretic model that was able to capture functional degradation in untrained SMAs and corroborated findings of 65 experimental specimens, as they demonstrated how both residual strain accumulation and transformation stress reductions examined at a system level affect performance, e.g., residual story drift for SMA-braced frames. The model is computationally efficient with an uncomplicated structure, so it can be easily utilized by practitioners who will not necessarily have a materials science background. Future developments of the model suggested include thermal reliance, shape memory effects, and low-cycle fatigue, so the appropriateness of the model for SMA based seismic devices can be further determined and refined.

In 2023, Yang et al. [61] gave a polynomial constitutive model using classical plasticity kinematic hardening. The authors used odd polynomial functions to make parameter identification simple but have a consistent tangent stiffness formulation to improve numerical convergence. Overall, the polynomial model is appropriate to reflect pseudoelasticity and shape memory behavior. A consistent tangent stiffness formulation was constructed to handle the variation in elastic modulus during phase transformation to help numerical convergence. Semi-implicit midpoint integration was used for numerical implementation. Validation against experimental data and previous models showed that the proposed model reasonably represented the pseudoelasticity and shape memory effects from SMA wires, and that it had good scalability for extensions to mixed hardening and large strain cases.

Alvares et al. [62] used the Preisach model. The authors developed the Everett functions from experimental data to describe SMA hysteresis effectively and generalized their functions to describe other macroscopic behaviors. They successfully achieved a clear data-driven advantage over classical phenomenological models or thermodynamic based models. An advantage of this approach is the straightforward numerical implementation is simpler than the phenomenological and thermodynamic-based models, which allow SMA behavior to be extricated beyond experimental results. These results were validated well against not only experiments conducted for this research, but also against literature data, and show that the Preisach model may be able to efficiently describe the macroscopic thermomechanical behaviors of SMAs.

Reddy and Maniprakash [63] extended SMA modeling to springs in torsional loading (helical springs). The authors proposed a multi-yield surface representation of the helical springs (that allowed for nonlinear moments) to describe inner loop hysteresis, pseudoelasticity, and temperature dependent strain recovery with torque and applied twist angle as state variables. The model uses torque and twist angle as state variables, with an internal variable to account for hysteresis. A non-iterative procedure makes the procedure computationally efficient. The framework can simulate both pseudoelastic and shape memory responses, including temperature dependent strain recovery and double hysteresis behaviors. They have also introduced a multi-yield surface approach to model the inner loop behavior. Limitations of the model include the inability to represent hysteresis between strain and temperature, suggesting future work in partial phase transformation scenarios and torsional fatigue analysis.

**Figure 3:**
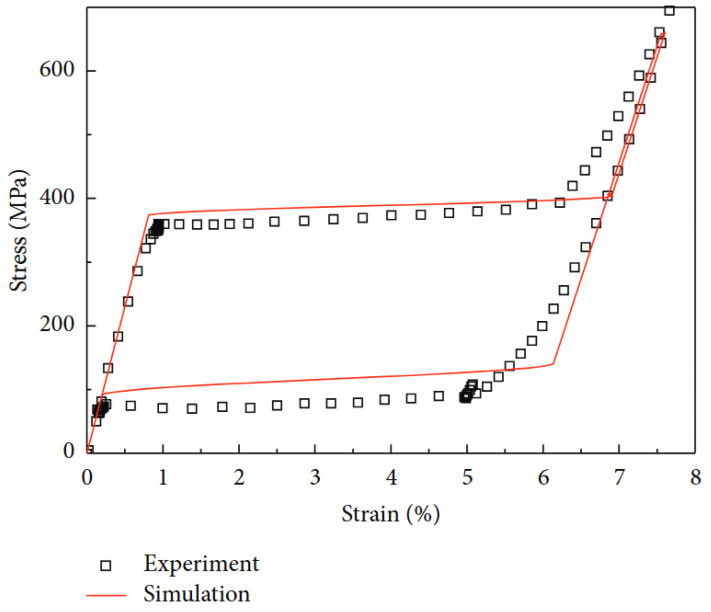
Prediction of superelastic behavior [64].

Roh [64] conducted a review on strain-rate effects on pseudoelastic SMA wire and proposed a simple 1D incremental thermomechanical model. The proposed model included various sets of nonconstant parameters, which also provided an acceptable fit to the stress–strain curves, and the relative heating of the wire that occurred, in a useful modeling framework for design analysis of an SMA cleverly based damping device. Overall, this compilation of work allows for an illustration of the evolution of SMA phenomenological models from simple pseudoelasticity to phenomenological models with rate effects, functional degradation, torsion response, uncertainty propagation, and numerical models that can be scaled. Figure 3 presents the ability of the model with thermomechanical parameters to predict the superelastic behavior of SMA wire.

Both experimental and numerical analyses demonstrated that the phase transformation stresses and the slope of the transformation plateau are dependent on increased strain rates, with minimal temperature variations. The numerical model provides a good prediction of the pseudoelastic stress–strain curves for varying strain rates, it is easy to implement, and it can help facilitate the design of damping devices that exploit the pseudoelastic behavior of SMAs.

### 4.2. Applications of 1D Phenomenological Models

**Figure 4:**
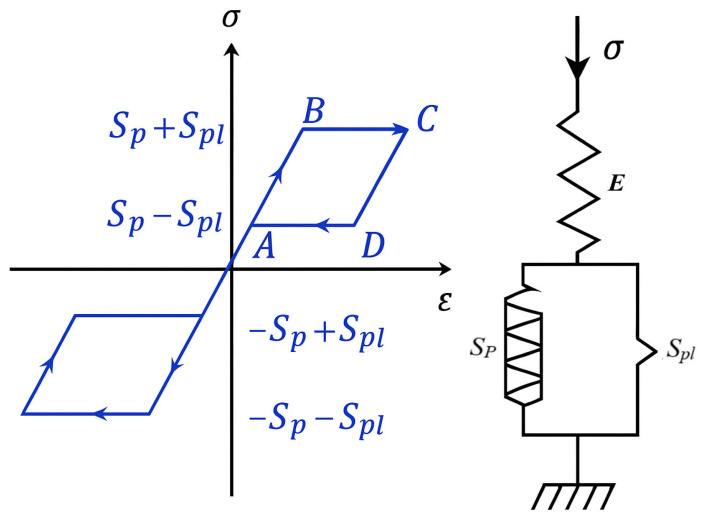
Rheological model of SMA material and its hysteretic loop (rearranged from [65]).

In addition to exclusively thermomechanical applications, many SMA-based devices operate in a multiphysics coupling mode, meaning that actuation and energy conversion are coupling more than one physical field. Some examples are SMA actuators that couple thermomechanical behavior with electrical resistance feedback for sensing and control, electromagnetic actuation mechanisms, or energy harvesting systems that transform thermal or mechanical energy into electrical output. These applications demand a modeling framework that accounts for interactions between phase transformation, thermodynamics, hysteresis, and electrical or electromagnetic effects. Most existing studies on SMAs rely on classical 1D phenomenological models to describe their thermomechanical response. More recent research, however, has introduced multiphysics-based formulations to improve predictive capability and enable functional design. To remain relevant in applications using SMAs in robotics, biomedical devices, space mechanisms, and energy harvesting, multiphysics has to be part of modeling SMAs in conjunction with 1D models. Initial works such as Zbiciak [65] provided the rationale for dynamic analysis of pseudoelastic SMA beams by creating a defined initial-boundary-value problem for Bernoulli–Euler beams by using a one-dimensional constitutive model that was derived from rheological principles as in Figure 4 and non-smooth mechanics. Zbiciak showed that by spatially discretizing the model and using the Runge–Kutta method (in MATLAB Ver. 7) to solve the resulting system of ordinary differential equations, the model sufficiently simulated hysteretic pseudoelasticity, giving the ability to create user-defined SMA elements in commercial finite element codes and offering an adaptable method to describe nonlinear SMA beam mechanics. In addition, they extended the SMA modeling to a biomedical application, proposing NiTi superelastic-antagonistic beams for smart pedicle screws. The fundamental behavior of the proposed beams was developed in a 3D finite element framework in ABAQUS Ver 6.7, where shape memory effect, superelasticity, and hysteresis were simulated. They demonstrated that the proposed SMA-based devices could be integrated with bidirectional loads for application to osteoporotic bones and highlighted the importance of geometric and material parameters to reliably integrate SMA devices in the biomedical field.

In their research, Sedlák et al. [66] concentrated on NiTi wires under combined tension, torsion, and bending conditions. They identified limitations in existing phenomenological models while developing a fully validated one-dimensional thermomechanical model of a NiTi wire assembly that designers could incorporate into more sophisticated designs. The model set the stage for improvement in fidelity and application of SMAs and their design in other microdevices, stents, and textiles.

Banerjee [67] advanced the modeling of SMA wire actuators by proposing a new algorithm accounting for phase kinetics that predicted wire behavior for arbitrary thermomechanical loading utilizing a single-state observer that demonstrated effectiveness in predicting both single wire configurations and antagonistic wire configurations. In the first scenario, the simulation results clearly indicate how discrepancies that were observed earlier are understood and how the algorithm can effectively reproduce SMA behavior with stress and temperature. The model is particularly successful in demonstrating the interruption and resumption of phase transformation in the same transformation zone. In the second case, the results show qualitative agreement with reported experimental results, thereby confirming that the algorithm is reliable. In general, these simulations demonstrate that the approach they have proposed can produce valid results for general thermo-mechanical loading of SMAs.

In 2013, the effects of loading rate on the seismic response of superelastic SMA-based devices were studied by Zhu and Zhang [68]. Three constitutive models (a rate-dependent thermomechanical model and two rate-independent phenomenological models) were evaluated via time-history analyses. The authors found that the rate-independent models, if calibrated with dynamic test data, performed very well in predicting peak displacements, ductility ratios, and accelerations, and while the differences in energy dissipation were more sensitive to hysteresis shape than loading rate, it is suggested that for many structural applications, simplified or rate-independent SMA models are generally sufficient, although when environmental temperature variations are likely significant, it was concluded that more sophisticated thermomechanical models would be important to incorporate.

The nonlinear free vibration of moving hybrid composite beams that integrated shape memory alloy fibers was studied in 2014 by Ebrahimi and co-workers. The recovery stresses from SMA fibers were obtained through the 1D Brinson model as well as the Reuss scheme. Nonlinear equations of motion were derived assuming that the beams behaved according to Euler–Bernoulli beam theory and invoked von Karman-type nonlinearity, and were solved analytically for simply supported beams. Parametric studies revealed that recovery stress, fundamental frequency, and buckling temperature were significantly affected by temperature, SMA volume fraction, pre-strain, and beam velocity. The study illustrated the ability of SMA fibers to enhance the dynamic performance of hybrid composite beams, and opened opportunities for further studies of the 1D SMA beam studies to include complex moving and nonlinear systems [69].

Samadpour et al. [70] expanded their work to thermally buckled SMA reinforced sandwich plates, and considered the implications of fiber placement and prestrain on dynamic response and post-buckling performance. They also proposed an analytical approach for bending of an SMA beam under thermal and pseudoelastic conditions. These results provide insights for the design of SMA-reinforced composite plates under thermal and mechanical load.

Ostadrahimi et al. [71] demonstrated a polynomial approximation for shear and deflection of SMA beams, and identified zero-stress fibers as a defining characteristic at low temperatures. The study simplified its 3D constitutive model to a 1D form, so that it could investigate the bending response of beams with symmetric cross-sections and with symmetric tension-compression behavior within a single analytic framework. The analysis for beams with rectangular cross-sections found relatively good agreement with both finite element analyses and experimental data. The study highlighted that zero-stress fibers appeared during unloading at low temperatures, which provided a deeper physical understanding of the bending behavior of SMA beams. Overall, the proposed analytical approach provided the capability to reduce computational cost while providing acceptable accuracy on both pure and general bending problems.

Viet, Zaki, and Umer [72] initiated analytical models of cantilevered superelastic SMA beams, tracking phase transformation evolution during loading–unloading cycles via moment–curvature and shear–strain relations to offer efficient alternatives to more complex design methods for SMA actuators, energy harvesters, and seismic energy dissipaters, while still mapping out phase transformation paths in detail. The relationship between bending moment and curvature was derived along the beam axis, and the beam was divided into three sections for pure austenite, pure martensite, and mixed phases. The analytical predictions of deflection and stress distribution were in excellent agreement with both uniaxial numerical models and FEA simulations. In 2018, extending this work, the authors [73] developed an analytical model–mathematical model based on Timoshenko beam theory- for superelastic SMA prismatic cantilever beams. In this model development, they acknowledged that in a loading–unloading cycle, phase transformation evolves in both transverse and longitudinal directions, and provided analytical relationships for moment–curvature and shear force–shear strain. The analytical model was verified against the numerical solution, showing very good agreement, while also providing the capability of direct calculation of the phase boundaries (as opposed to post-processing through FEA). Compared to previous developments based on Euler–Bernoulli considerations, this work was a significant advance—also particularly evident and important for dynamic specifications such as robotic actuation, use of SMA beams for seismic energy dissipation, and energy harvesting.

Bin Huang, Hongwang Lv, and Yang Song [74] used experimental and numerical methods to investigate the behavior of superelastic SMA helical springs as a simplified iterative force-displacement relationship for complex loading and unloading. To accommodate complex loading and unloading, the authors utilized a modified multilinear constitutive model based on the 1D Motahari model by modifying the stress–strain curves to match the experimental data. The authors were able to consider both torque and bending moments from the SMA wire cross-sections and observe realistic spring behavior with respect to displacement and load. The experimental studies performed on four NiTi helical spring specimens demonstrated stable superelasticity, good self-centering capabilities, and a consistent recentering ability. The results indicated that the proposed model accurately replicated the dominant hysteresis loops and subloops observed in complex loading situations, and that the model remained accurate through moderate ranges in temperature. Overall, the authors noted that the model was suitable for evaluating the force-displacement behavior of SMA springs, and proposed future work for developing SMA-based sensors for displacement, or force, with good accuracy.

**Figure 5:**
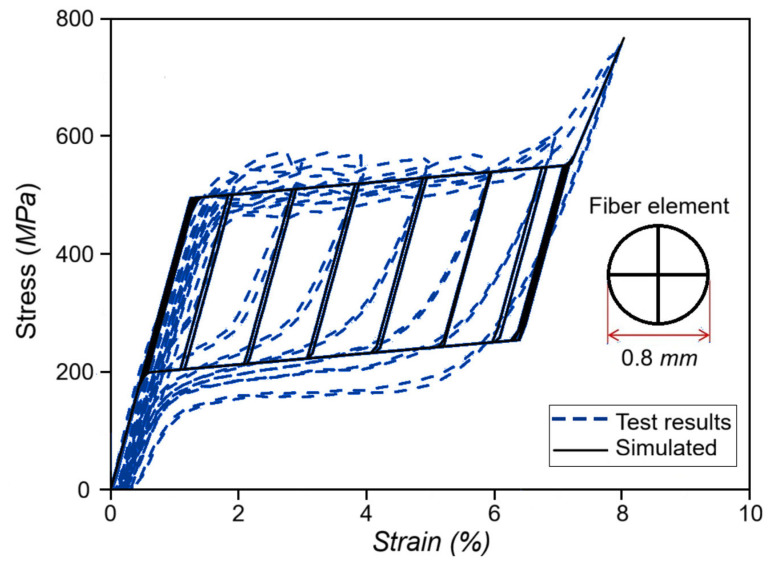
Comparison of numerical simulation with test results (rearranged from [75]).

Fang et al.’s [75] evaluations of NiTi SMA cables subject to cyclic loading, proposed that annealing treatment was a factor in controlling phase transformation, stiffness, energy loss, and self-centering, that may be applied to mitigate seismic events in bridges. An uncomplicated yet remarkable numerical model successfully represented the cables superelastic behavior. In a model bridge, using SMA cables as restrainers showed a meaningful reduction in peak and residual displacements, validating the use of SMA cables for seismic damage mitigation. Figure 5 shows stress–strain simulation has a good correlation with the test results for a typical Sma wire.

In Xiao and Jiang’s work [76], a new constitutive model was developed to describe the cyclic response of superelastic NiTi SMA wires. After running several cyclic tensile tests, the model was implemented in finite elements using a backward Euler integration scheme, included separate elastic features for the austenitic and martensitic phases, and constituted two distinct internal variables (transformation strain and residual strain). This overall model captured cyclic phenomena accurately, such as the accumulation of residual strain, lost dissipated energy, decreased transformation stress, increased transformation hardening modulus, and decreased reversible transformation strain. Furthermore, the model highlighted strain inhomogeneity because it captured the transformation pattern related to material softening during martensitic transformation. Simultaneously, the strain rate dependent transformation patterns in superelastic NiTi tubes (D/t = 10) undergoing uniaxial tension were studied experimentally and numerically. It was found that mechanical instability during the martensitic transformation is expressed as inhomogeneous deformation and a stress plateau that evolves with strain rate; for example, a horizontal plateau with cylindrical transformation bands evolves into a hardening plateau with helical bands with increasing strain rate. The evolution response was explained in terms of mechanically favorable cylindrical bands at low strain rates competing with thermomechanically favorable helical bands at higher strain rates, providing new insights into the interactions between strain rate, instability, and transformation patterns in phase-transformable materials [77].

Within actuator design, Bhatt et al. [78] examined SMA spring actuators under two biasing schemes: a steel spring and an SMA wire. They reported that the SMA spring–wire configuration has comparable deflection while showing higher controllability, greater degrees of freedom, and better actuation performance. Xiao and Jiang [79] evaluated buckling and unbuckling of NiTi tubes, demonstrating the effects of geometry and phase transition on column- and shell-type buckling, which can assist in the design of anti-buckling SMA components.

Ahčin et al. in 2022 [80], developed a one-dimensional numerical model to simulate and optimize a shell-and-tube-like active elastocaloric regenerator (AeCR) constructed of NiTi tubes. The superelastic and elastocaloric behavior of NiTi was experimentally characterized and used as input into a phenomenological model. The model included three domains: elastocaloric material, heat transfer fluid, and housing. Hysteresis losses were accounted for as entropy irreversibilities when loading and unloading. Comparison to actual experimental data showed good agreement on temperature span, cooling/heating power, and COP to validate that the model should be effective for performing performance optimization of shell-and-tube-like AeCRs.

Rezaee-Hajidehia and Rý [81] studied return-point memory in nested subloop deformation of NiTi SMAs and created a gradient-enhanced phenomenological model of functional fatigue that considers the complex coupling of local martensitic instabilities with transformation-enhanced plasticity and cyclic degradation. Further work on NiTi strips possessing more complex transformation sequences showed the entangled development of phase transformation and transformation-induced plasticity under multiple loading programs, and also the contribution of thermomechanical coupling effects. This work underscored the importance of localized phase transformation instabilities for cyclic functional degradation, often overlooked in previous modeling.

Annadata et al. [82] validated the Woodworth and Kaliske SMA model in a method that allows for interaction of SMA wires in fiber–rubber composites. They showed that the predictability of the model is valid for U-shaped SMA wires and highlighted the importance of spatial configuration and pre-strain for predicting bend-twist coupling, with applications in adaptive systems.

**Figure 6:**
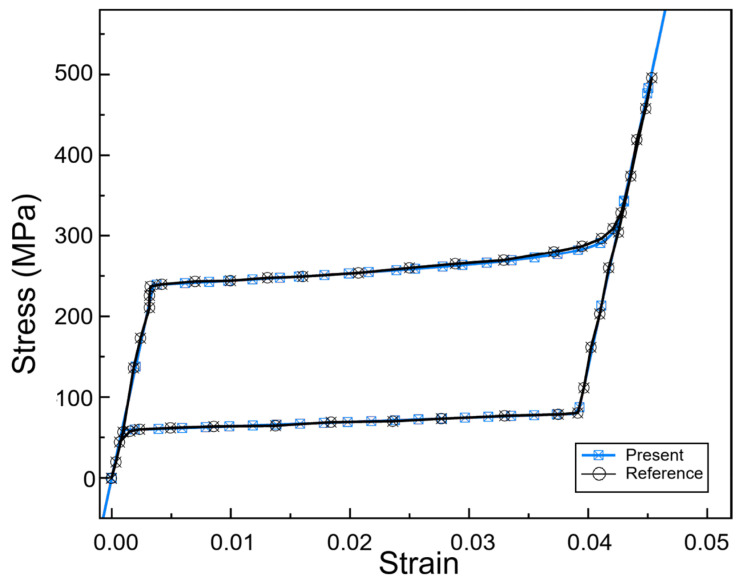
Validation of stress–strain relation with reference data available in the literature (rearranged from [83]).

Zhang and Semperlotti [83] considered the use of SMA periodic beams related to elastic wave propagation, and noted that pseudoelastic behavior and level of pre-strain may be useful for broadband wave attenuation. Using a reduced 1D Panico-Brinson (RPBM), they demonstrated that lattices with random and functionally graded distortions allow for further tailoring of energy dissipation, while utilizing properties that are well established in periodic structures of SMAs. This model was verified with the existing references, as in Figure 6. All in all, findings illustrate the potential for SMAs in the vibration control space, using the designed (and coupled) deformation and softening behavior of SMAs, or to team SMA periodic structures with sufficiently flexible structural elements of appropriate scale, for analogous applications.

## 5. Open Challenges and Future Directions

The studies on shape memory alloys provide a number of open challenges and areas for future work, which can be categorized based on our work into two broad areas: phenomenological modeling and application.

With respect to modeling, despite the remarkable development of 1D phenomenological constitutive models for SMAs over the past two decades, several open challenges remain that limit their predictive validity and applicability. One of the foremost limitations is the treatment of strain-rate and frequency effects. Models proposed by Zhu and Zhang [48], Qian et al. [54], and Roh [64], for instance, include rate dependencies, but the constitutive laws are ultimately based on non-comprehensive assumptions that do not consider the entire threat of thermomechanical coupling in a given dynamic or high-frequency loading.

Another ongoing weakness is in representing functional degradation and fatigue effects. Even though recent works, including Lee and Jeon [60], developed residual strain accumulation and transformation stress reduction models and these models, are beneath a parametric and empirical model and correspond to quad-specific alloy composition or training history, a rational model to necessarily generalize degradation across alloys, loading history, and environmental conditions is not demonstrated. Developing models is crucial to predict the long-term performance, safety assurance, critical safety in seismic damping and biomedicine devices, etc.

An additional challenge is the counting and propagation of uncertainty. Oliveira, Savi, and Santos [58] specifically pointed out the sensitivity of SMA model predictions to elastic moduli and change in temperature, but uncertainty treatment is often not explicitly included in most phenomenological models. With the increasing emphasis on reliability-based design, future 1D models need to take probabilistic approaches to uncertainty quantification and approaches to link laboratory characterization to a real application.

Also, under a complex thermomechanical loading path, today’s 1D models still have significant limitations. Models such as those by Reddy and Maniprakash [63] and Marfia and Rizzoni [57] extended classical models to consider torsional springs and tension–compression asymmetry, respectively, but there still are no general formalisms that can replicate multiaxial-like effects in a concise 1D model. The next step is to improve the descriptive capability of simpler 1D formulations while maintaining simplicity and computational efficiency.

Another emerging path is fusing data-driven techniques into phenomenological modeling. Alvares et al. [62] have shown the promise of using Everett functions from experimental data to better capture hysteresis than phenomenological models. Creating hybrid models may utilize traditional phenomenology with machine learning or system identification approaches to create more adaptive, generalizable, and scalable 1D models in contexts with available experimental data.

Ultimately, numerical robustness and convergence remain an empirical barrier for many 1D realizations, especially when endeavored within commercial finite element or similar codes. Although there are conceptual reductions like polynomial iterated fits (Yang et al. [61]) that could assist with parameter identification and ultimately convergence, applying models like these creates further complications when embedded into large-scale structural simulations. Future work should extend focus onto numerically stable numerical formulations while retaining physical equivalency and limiting computational effort (and thus allowing for mitigation of uncertainties), so they can be applied much more quickly during engineering design processes.

In conclusion, the future of the 1D phenomenological SMA models requires rate-dependent formulations, systematic models of functional deterioration, direct integration of uncertainty quantification with phenomenological modeling, extending simplifications to capture behaviors with greater degrees of load, and encouraging data-driven hybrid approaches to research and refine phenomenological characterization, while improving numerical robustness. Addressing challenges in these areas will help ensure that 1D phenomenological models continue to serve as an effective bridge between understanding fundamental material behavior and its application in practical engineering design applications, especially where computational simplicity must coincide with productive accuracy.

On the application side, whereas immense progress in recent decades has been made in applying one-dimensional SMA modelings in structural, biomedical, actuating, and vibratory control applications, several issues remain unraveled, which limit their full potential in such applications. One such issue is in converting idealized 1D modelings into real device geometrical and loading scenarios. Such papers as those by Sedlák et al. [66] and Banerjee [67] presented the success of validated 1D thermomechanical modelings for wire and actuating applications, but when extended into applications involving biomedical devices or structural members, geometric nonlinearities, multi-axial state-of-stress conditions, and inhomogeneously varying temperature fields often make predictive accuracy unsatisfactory. Future advances should focus on refined scaling schemes that can maintain 1D formulation simplicity while in a systematic way incorporating geometric effects and boundary conditions in order to close the analytical versus actual device behavior gap.

Another open question is in strong modeling of compression–tension asymmetry, cyclic degradation, and fatigue behavior in application-driven conditions. Though superior formulations by Xiao and Jiang [76] addressed asymmetry and cyclic instabilities, application-driven research still retains mostly rate-independent or phenomenologically calibrated parameters unable to continuously predict long-term deterioration subjected to repeated excitation. For applications such as seismic dampers, medical implants, or SMA-type spring units, deterioration in hysteresis loops or residual strain accumulation is not a side effect but a sign of service life. Creating practical, and accurate degradation-aware 1D constitutive models is an urgent direction for advancing reliable application under long-term cyclic loading.

Integration of SMAs into hybrid systems and multifunctional composites gives a dual effect: improve performance while increasing complexity. Work by Ebrahimi et al. [69], Samadpour et al. [70], and Annadata et al. [82] illustrated how SMA fibers/wires can be integrated with polymeric, metallic, or rubber matrices to give composites whose responses are tunable. However, 1D modeling in such a case tends to oversimplify SMA elements’ interaction with their host matrix, so there are mismatches when it is tried to predict load transfer behavior or debonding at interfaces or heat localization effects. Future work must develop hybrid modeling approaches in which 1D SMA modeling is combined with mesoscale modeling schemes for matrix–fiber contacts so that prestrain, geometrical arrangement, and environmental variation are taken as design variables.

Another critical issue is thermomechanical coupling and rate-dependent behavior of SMA in dynamic applications. Prior publications such as Zbiciak [65], Zhu and Zhang [48], and Zhang and Semperlotti [83] noted pseudoelastic damping and wave attenuation were heavily pre-strain level, straining-rate, and temperature-variant. Nonetheless, a majority of practical applications—with elastocaloric cooling to seismic energy dissipation still utilizing model descriptions assuming no localized heat onset or instantaneous heat transport—it remains an assumption that creates a gap between prediction results in a controlled lab environment versus in-service use where localized hotspot or heat gradient onset or even a rate-dependent instability can significantly alter device behavior. Consequently, an effective thermo-mechanically coupled 1D formulation accounting for heat transfer time scales, phase kinematics, and loading rates within a consistent framework is a required course for study in the future.

High computational cost and numerical stability in incorporating 1D SMA models within finite element analysis are still problematic. Largely theoretical works such as Zbiciak [65] and Frost et al. [55] demonstrated 1D SMA model implementation within MATLAB Ver. 7 or ABAQUS Ver. 6.7, but scaling such 1D SMA models for complete structural system simulation can result in convergence issues when cyclic or multi-branch hysteretic loading is applied. Polynomial approximation (Ostadrahimi et al. [68]; Yang et al. [61] in applications of modeling) reduced some such issues, but a general framework for a stable, efficient, user-friendly integration within a commercial program continues as an open issue. Such works involving reduced-order modeling, adaptive algorithms, and modular software approaches would perhaps fill such a gap and render SMA applications more practical for daily practitioners.

Overall, the use of hybrid and data-driven methods is an exciting option for future directions. Experimental work by Huang, Lv, and Song [74] discovered the complexity of SMA spring behavior, while Alvares et al. [62] showed in the modeling context that Everett function-based hysteresis models could produce better responses than conventional formulations of hysteresis. In regard to applications, if experimental databases are developed and incorporated in conjunction with machine learning and system identification, there would be possibilities to create predictive 1D models that would adjust for devices or conditions, ultimately providing both greater accuracy and ability to generalize. This could be beneficial for adaptive systems and smart structures because SMA elements would be subjected to diverse loadings that would not be adequately captured by models based on phenomenological analyses alone.

To summarize, by overcoming these barriers, 1D SMA models will not only continue to remain a pillar of application-based design, but will also grow as reliable predictors of next-generation adaptive and multifunctional engineering system performance.

Table 2 summarizes the challenges and future directions in two categories: modeling and applications.

## 6. Conclusions

The one-dimensional phenomenological modeling of shape memory alloys has developed from the seminal formulations of Tanaka, Liang–Rogers, and Brinson, to numerous recent developments in constitutive and application-related modeling. Each path of model development has sought to balance simplicity, computational efficiency, and continuity with the physical processes, resulting in models that begin to account for stress–strain–temperature relationships, phase transformation kinetic effects, and, more recently, rate dependence, functional degradation, and uncertainty propagation. By comparing the foundational models of SMAs, we highlighted the strengths and weaknesses of each model and showed that, while the earlier models are useful for conceptual design and control applications, the more recent models complemented the earlier forms by predictive capabilities that included cyclic effects, asymmetry, and thermomechanical couplings.

In parallel to theoretical developments, there has been extensive application of phenomenological models. Some of the applications encompass modeling for SMA beams undergoing dynamic analysis, biomedical applications including advanced pedicle screws, seismic damping systems, hybrid composites, and elastocaloric cooling systems. There is an array of works related to the application of 1D models as reasonable approximations for engineering design. The reviewed literature shows that simulation driven designs and innovations are achievable even with considerable geometrical and operational complexity. Simultaneously, the applications also demonstrate limitations of the model, especially under multi-axial and cyclic and fatigue loading conditions that would then require higher fidelity frameworks or hybrid models.

Future works in SMA modeling would benefit from a more consolidated and standardized formulation that reconciles the wide variety of phenomenological approaches. Future research should also focus on the improved representation of rate dependent behavior, cyclic degradation, and uncertainty quantification, as these are the primary impediments to model robustness and wider applicability. In addition, 1D models coupled with multi-axial and system-level formulations may prove to be an effective strategy for bridging fundamental advances with engineering applications and supporting a more reliable and scalable testing framework.

Despite these achievements, significant challenges remain. The treatment of rate effects, functional degradation in time (i.e., fatigue), and uncertainty accounting still limit the broad applicability of models. Moreover, one-dimensional systems are limited to uniaxial or quasi-uniaxial—a gap when bridging to real-life multi-axial systems. Some of these gaps could be addressed through interdisciplinary modeling paradigms that combine phenomenological modeling approaches with multiscale simulations, probabilistic frameworks, and the emergent use of data-driven predictive methods based on machine learning. In conclusion, this review has examined the evolution of phenomenological modeling of SMAs from foundational formulation to current applications while exposing significant gaps that define priorities for future research. By systematically comparing phenomenological formulations and documenting advances in modeling and applications, this review will serve as a base for researchers and engineers to refine existing frameworks, investigate new application areas, and hopefully drive the functional frontiers of SMAs in adaptive and intelligent systems.

In summary, the ongoing development of phenomenological SMA models, alongside standardized validation processes, will be the key to moving them from academic research to established engineering practices. With sustained collaboration in modeling, experimentation, and systems-level design, SMA technologies are well positioned for the next generation of adaptive, resilient, and intelligent material systems.

## Figures and Tables

**Table 1:** Evolution of basic phenomenological models.

Feature	Tanaka (1986) [41]	Liang and Rogers (1990) [42]	Brinson (1993) [43]
Main Contribution	First consistent thermomechanical phenomenological SMA model for uniaxial loading	Improved phase transformation kinetics and smooth transition description	Separated stress- and temperature-induced martensite fractions; enhanced modeling of SME and superelasticity
Constitutive Equation	Links stress to strain, temperature, martensite fraction	Extends Tanaka’s framework by incorporating more detailed internal variables and transformation kinetics, allowing a refined description of phase evolution.	Based on Tanaka’s but distinguishes ξ into stress- and temperature-induced parts
Key Internal Variable(s)	Martensitic volume fraction (ξ)	Martensitic volume fraction (ξ)	ξ divided into stress-induced ($\xi_{s}$) and temperature-induced ($\xi_{T}$) fractions
Phase Transformation Kinetics	Exponential functions for forward and reverse transformations	Cosine-based continuous functions for smoother transitions	Cosine-based, with $\xi_{s}\;$ and $\xi_{T}\;$ separated, better captures detwinning and SME below $M_{f}$
Elastic Modulus Variation	Linear interpolation between $E_{A}$ and $E_{M}$	Same as Tanaka	Same as Tanaka
Recoverable Strain	Transformation-influence coefficient Ω(ε)	Similar as Tanaka	Similar, but modified for separated ξ
Key Assumptions	Isothermal/uniaxial; simple kinetics	Uniaxial; refined transformation kinetics (hysteresis captured)	Uniaxial; distinguishes stress vs. temperature effects
Complexity	Low; easy to implement	Low to moderate; simple smooth kinetics	Moderate; additional variables, more calibration needed
Parameters Required	$E_{A}$, $E_{M}$, $\varepsilon_{L}$, transformation temperatures, kinetic coefficients	Same + cosine kinetics constants	Same + separate kinetic laws for $\xi_{s}$ and $\xi_{T}$
Practical Advantages	Very simple; good for basic SME/superelastic prediction	Improved hysteresis modeling; smoother phase transformation	Captures complex hysteresis; models detwinning and stress/temperature coupling more accurately
Main Limitations	Limited accuracy for complex cycles; oversimplified kinetics	Same as Tanaka; still uniaxial only	More parameters; still 1D; more calibration effort
Applications	Educational use and analytical studies; simple SMA actuator demonstrations	Improved actuator modeling; better hysteresis prediction; superelastic simulation	Actuator design, smart structures, SMA wires and rods under thermal cycling

**Table 2:** Open challenges and future directions in 1D SMA modeling and applications.

Category	Challenge	Current Status	Future Directions
Phenomenological Modeling	Strain-rate and frequency effects	Rate-dependent models exist (Zhu & Zhang [48], Qian et al. [54], Roh [64]), but thermomechanical coupling under dynamic loading is limited	Develop comprehensive rate-dependent models considering dynamic and high-frequency thermomechanical coupling
	Functional degradation and fatigue	Empirical models exist (Lee & Jeon [60]) but alloy- and history-specific	Systematic models generalizing degradation across alloys, loading history, and environments
	Uncertainty quantification	Sensitivity noted (Oliveira et al. [58]); most models ignore explicit uncertainty	Integrate probabilistic approaches and link lab characterization to real applications
	Multiaxial effects under complex loading	Extensions exist (Reddy & Maniprakash [63], Marfia & Rizzoni [57])	Improve 1D formulations to capture multiaxial-like behaviors while maintaining simplicity
	Data-driven and hybrid modeling	Everett-function approaches show promise (Alvares et al. [62])	Combine phenomenology with machine learning or system identification for adaptive and scalable models
	Numerical robustness and convergence	Polynomial fits help (Yang et al. [61])	Develop numerically stable formulations suitable for large-scale simulations
Applications	Translating idealized 1D models to real geometries	Wire/actuator applications validated (Sedlák et al. [66], Banerjee [67])	Refined scaling schemes to incorporate geometry, boundary conditions, and thermal effects
	Compression–tension asymmetry and cyclic degradation	Advanced formulations exist (Xiao & Jiang [76]), mostly phenomenological	Create degradation-aware 1D models for long-term cyclic loading in practical devices
	SMA in hybrid/multifunctional composites	SMA–matrix interaction oversimplified (Ebrahimi et al. [69], Samadpour et al. [70])	Develop hybrid modeling combining 1D SMA with mesoscale matrix–fiber interaction models
	Thermomechanical coupling in dynamic applications	Lab-based pseudoelastic damping studies (Zbiciak [65], Zhu & Zhang [48])	Effective 1D models accounting for heat transfer, phase kinetics, and rate-dependent effects
	Computational cost and stability in FE analysis	MATLAB/ABAQUS implementations demonstrated (Zbiciak [65], Frost et al. [55])	Reduced-order modeling, adaptive algorithms, modular software for stable, efficient integration
	Hybrid and data-driven applications	Everett-function and experimental databases show potential (Huang et al. [74], Alvares et al. [62])	Predictive models integrating machine learning and system identification for adaptive SMA-based devices

## Data Availability

The data that supports the finding of this study are available from the corresponding author upon reasonable request.

## Abbreviation

The following abbreviation is used in this manuscript:

SMA	Shape Memory Alloy
